# Aortoduodenal syndrome caused by infrarenal abdominal aortic aneurysm: Evolution of CT findings and early imaging clues

**DOI:** 10.1016/j.radcr.2026.07.122

**Published:** 2026-08-26

**Authors:** Alex Daniel Medina-Siosi

**Affiliations:** Department of Radiology, Katholisches Karl-Leisner Klinikum, Kleve, Germany

**Keywords:** Abdominal aortic aneurysm, Duodenal obstruction, Tomography, X-ray computed, Superior mesenteric artery syndrome, Endoleak

## Abstract

Aortoduodenal syndrome is a rare cause of proximal intestinal obstruction resulting from extrinsic compression of the third portion of the duodenum by an abdominal aortic aneurysm. We report the case of a 59-year-old man with longstanding early satiety and progressive weight loss in whom an infrarenal abdominal aortic aneurysm was initially detected incidentally. Despite multiple imaging studies, the characteristic computed tomography (CT) features of clinically significant duodenal obstruction did not become apparent until 2026, when contrast-enhanced CT demonstrated marked proximal duodenal dilatation with an abrupt transition at the third portion caused by aneurysmal compression. Retrospective review of earlier CT examinations demonstrated subtle imaging findings that were insufficient to establish the diagnosis at the time but preceded the development of overt imaging evidence of obstruction. This case highlights the importance of correlating serial CT findings with the clinical presentation and emphasizes that proximal duodenal dilatation together with an abrupt transition point are the key imaging features supporting the diagnosis of aortoduodenal syndrome. Recognition of these findings may facilitate earlier diagnosis and improve patient management.

## Introduction

Abdominal aortic aneurysm (AAA) is a common vascular condition encountered on cross-sectional imaging and is associated with a wide spectrum of complications, including mass effect on adjacent structures [1,2]. Although most complications are well recognized, extrinsic compression of the gastrointestinal tract is rare and often underrecognized [2,3].

Aortoduodenal syndrome is an uncommon cause of proximal intestinal obstruction resulting from compression of the third portion of the duodenum by an abdominal aortic aneurysm [3,4]. Its clinical presentation is often nonspecific, which may lead to delayed or missed diagnosis [3,5].

The main differential diagnosis is superior mesenteric artery syndrome, in which duodenal compression occurs between the aorta and the superior mesenteric artery due to a reduced aortomesenteric angle, typically in the setting of significant weight loss [6]. Despite similar clinical presentations, the underlying mechanisms differ and have important implications for management.

Computed tomography (CT) plays a central role in the evaluation of these entities by allowing accurate assessment of vascular anatomy and duodenal compression [2,4]. Recognition of key imaging features is essential to establish the correct diagnosis and guide appropriate treatment.

In this report, we describe a case of aortoduodenal syndrome diagnosed on contrast-enhanced CT in a patient with longstanding early satiety and progressive weight loss. Retrospective review of earlier CT examinations demonstrated subtle imaging findings that were insufficient to establish the diagnosis at the time but became meaningful as the characteristic features of duodenal obstruction evolved.

## Case presentation

A 59-year-old man presented to the emergency department in October 2017 with intermittent upper abdominal pain, early satiety, and abdominal fullness. Physical examination and abdominal ultrasound incidentally revealed an infrarenal abdominal aortic aneurysm. Upper gastrointestinal endoscopy demonstrated duodenitis.

Contrast-enhanced CT angiography performed on October 27, 2017 confirmed an infrarenal abdominal aortic aneurysm measuring approximately 4 cm in maximum diameter. Given the aneurysm size and the patient’s clinical stability, conservative management with imaging surveillance was recommended. A follow-up CT performed 1 week later showed no acute abdominal abnormalities requiring further intervention. As the patient's abdominal pain partially improved following medical treatment for duodenitis, he was discharged with outpatient follow-up.

Over the following years, abdominal pain and bloating gradually improved; however, early satiety persisted. The patient reported markedly reduced oral intake due to rapid fullness after small meals despite preserved appetite, resulting in progressive weight loss from 76 kg to 57 kg over approximately 5 years.

**Fig. 1:**
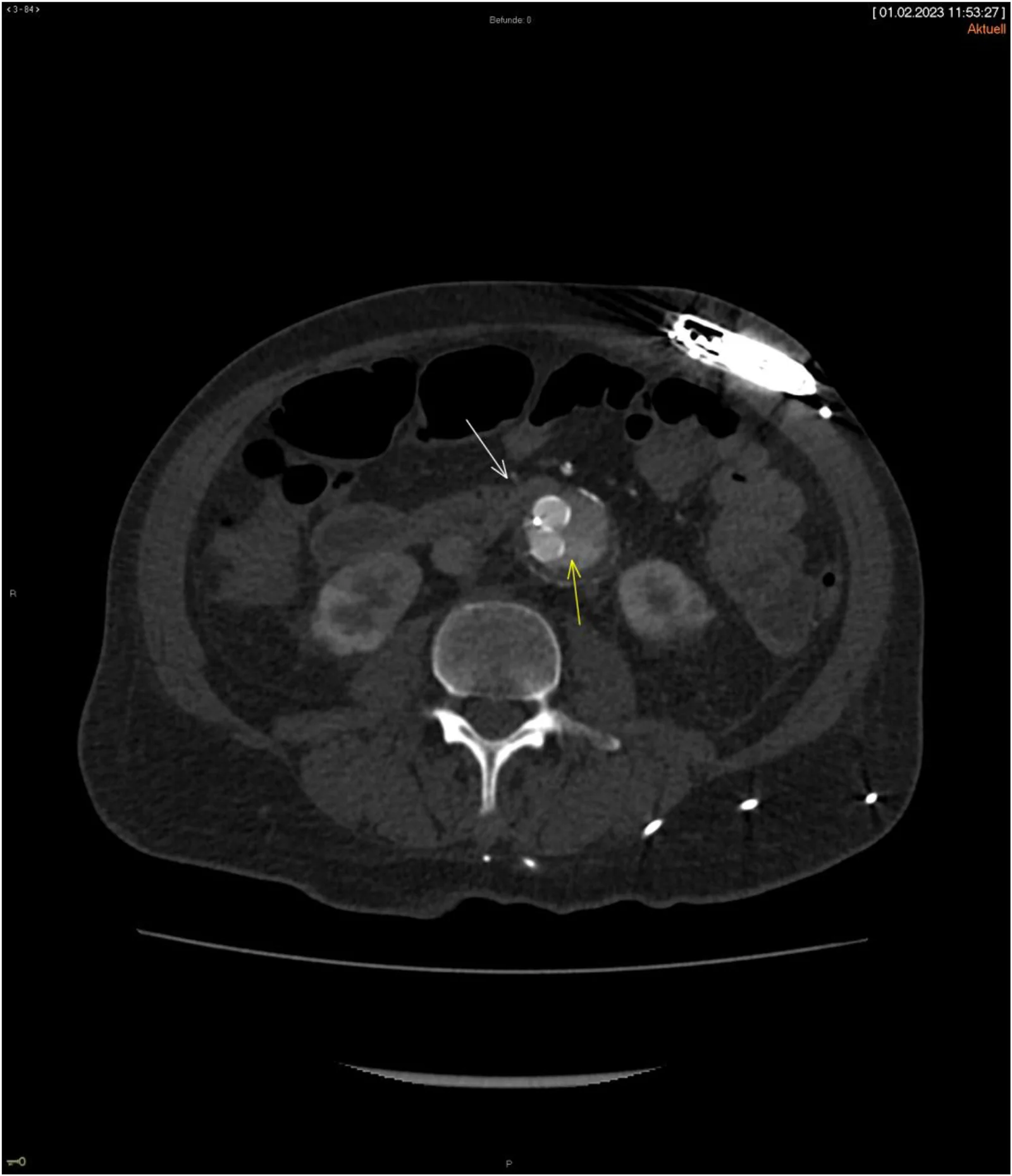
Post-EVAR axial contrast-enhanced CT image obtained at the referring institution demonstrating an endoleak within the aneurysm sac (yellow arrow). Focal narrowing of the adjacent third portion of the duodenum is also present (white arrow), without convincing upstream duodenal dilatation on the available image.

Serial follow-up imaging performed at the referring institution demonstrated progressive enlargement of the aneurysm, and endovascular aneurysm repair (EVAR) was performed on January 31, 2023 after the aneurysm reached the institutional threshold for intervention. The imaging study documenting aneurysm enlargement was performed at the outside institution and is not available in the present report. On the first postoperative day, the patient developed back pain, flank pain, and recurrent upper abdominal discomfort. Review of postoperative CT performed at the referring institution demonstrated an endoleak (Fig. 1) together with a wedge-shaped cortical perfusion defect involving the lower pole of the right kidney, interpreted in the original report as compatible with renal infarction. Details regarding endoleak classification and subsequent management were not available for review.

Although postoperative symptoms partially improved, early satiety and intermittent upper abdominal discomfort persisted. The patient did not seek further medical evaluation for these chronic gastrointestinal symptoms.

**Fig. 2:**
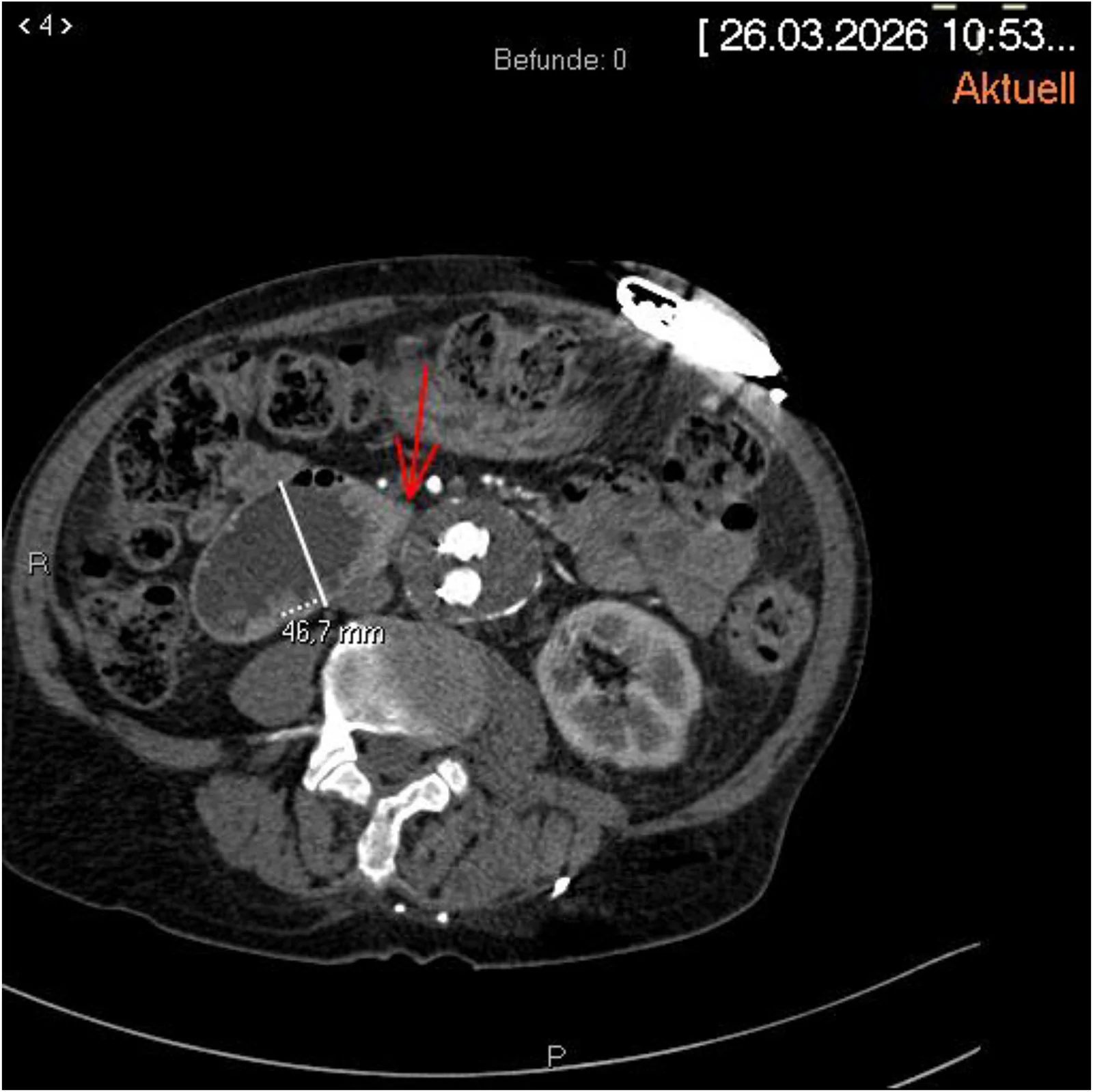
Axial contrast-enhanced CT image from 2026 demonstrating marked upstream duodenal dilatation, measuring up to 46.7 mm, with an abrupt transition at the third portion of the duodenum (red arrow) adjacent to the infrarenal abdominal aortic aneurysm, consistent with aortoduodenal syndrome.

**Fig. 3:**
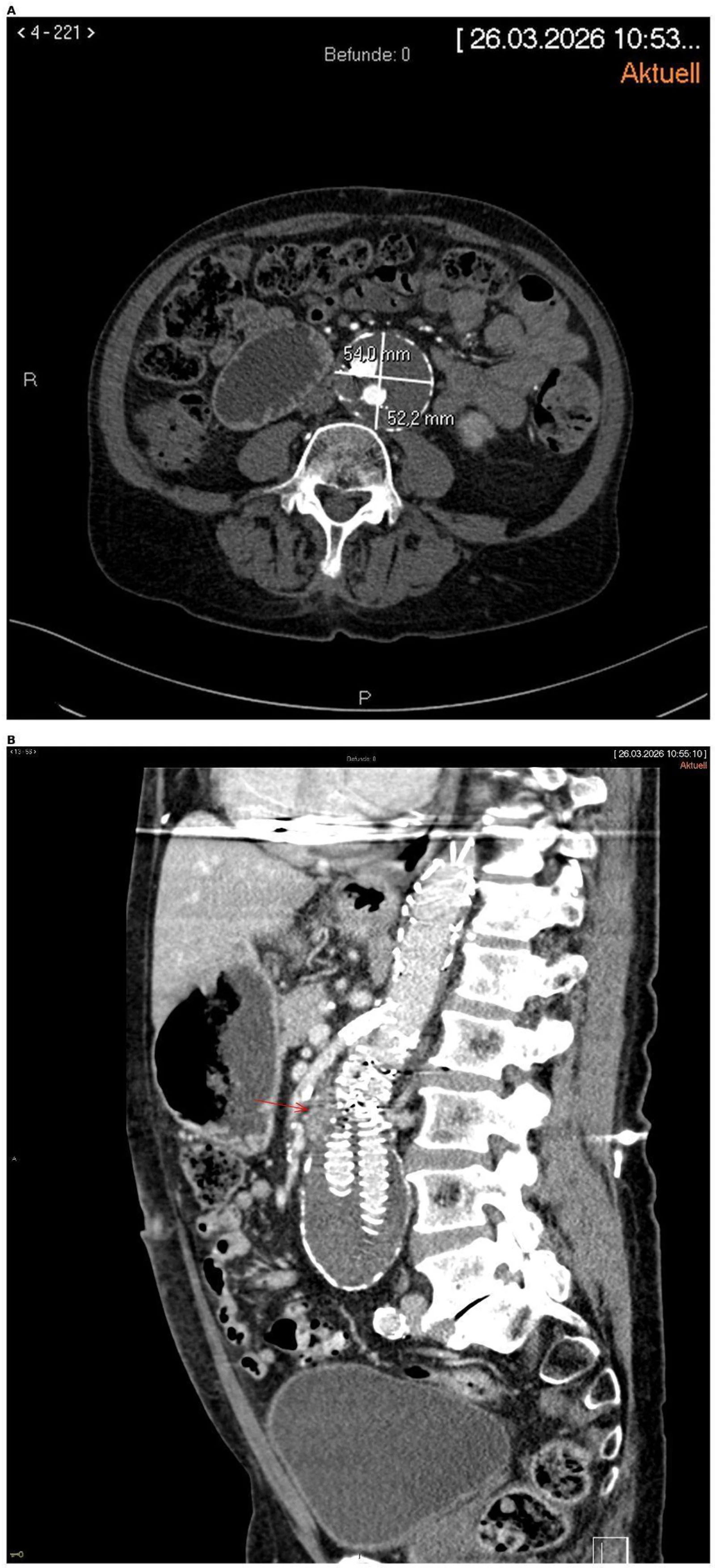
Axial (A) and sagittal (B) contrast-enhanced CT reconstructions from 2026 demonstrating the anatomical relationship between the infrarenal abdominal aortic aneurysm and the third portion of the duodenum. The aneurysm measures approximately 51 × 52 mm (A). The sagittal reconstruction (B) demonstrates focal compression of the third portion of the duodenum at the level of the aneurysmal sac (red arrow).

In 2026, the patient presented to our institution after a new-onset seizure. Because of his marked weight loss, progressive clinical deterioration, and the absence of an established explanation for these findings at that time, contrast-enhanced CT of the chest and abdomen was performed to exclude an underlying malignancy. CT demonstrated marked dilatation of the proximal duodenum with an abrupt transition at the third portion, where extrinsic compression by the infrarenal abdominal aortic aneurysm was evident despite previous EVAR (Fig. 2, Fig. 3). These imaging findings, together with the patient's longstanding clinical symptoms, established the diagnosis of aortoduodenal syndrome.

**Fig. 4:**
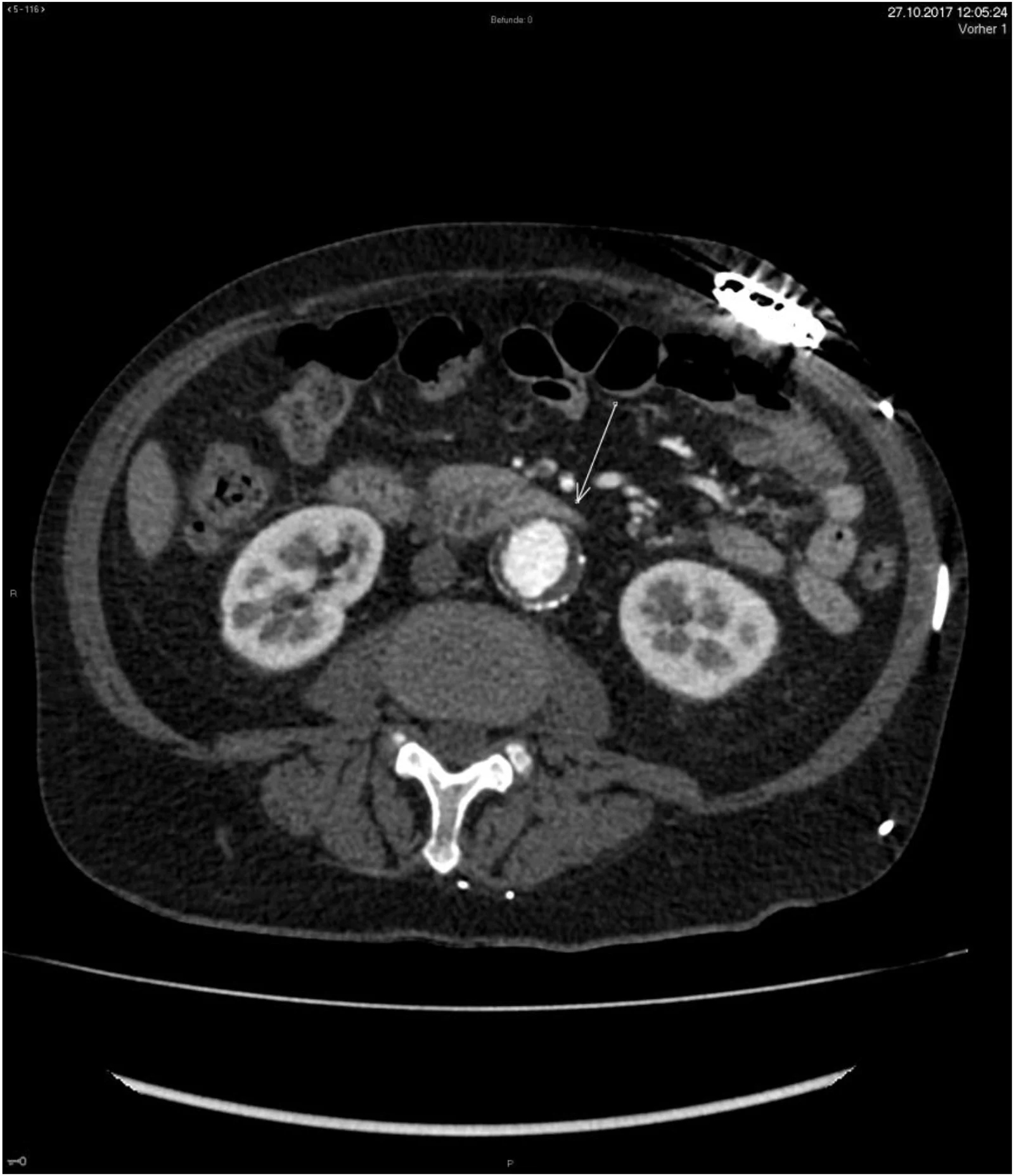
Retrospective axial contrast-enhanced CT image from 2017 demonstrating focal narrowing of the third portion of the duodenum adjacent to the infrarenal abdominal aortic aneurysm (arrow). No convincing upstream duodenal dilatation was present, and the imaging findings were insufficient to establish a diagnosis of clinically significant duodenal obstruction at that time.

Retrospective review of the previous CT examinations obtained from the referring institution demonstrated subtle contact between the aneurysm and the third portion of the duodenum; however, convincing upstream duodenal dilatation was not present on the earlier studies (Fig. 4). In retrospect, these findings were considered suggestive of evolving extrinsic compression but were insufficient to establish the diagnosis at that time.

Repeat upper gastrointestinal endoscopy again demonstrated duodenitis. No intraluminal obstructing lesion was reported. The patient’s acute neurological presentation was ultimately attributed to neuroborreliosis. After multidisciplinary discussion, treatment options—including the potential risks associated with further surgery following previous EVAR—were explained to the patient. Given the chronic nature of his symptoms and the complexity of additional surgical intervention, the patient elected conservative management with clinical follow-up.

## Discussion

Aortoduodenal syndrome is a rare cause of proximal intestinal obstruction resulting from extrinsic compression of the third portion of the duodenum by an abdominal aortic aneurysm [[3], [4], [5]]. Although abdominal aortic aneurysms are frequently encountered on cross-sectional imaging, gastrointestinal obstruction due to aneurysmal mass effect remains uncommon and is likely underrecognized because of its nonspecific clinical presentation [1,3].

Patients typically present with chronic upper gastrointestinal symptoms, including nausea, vomiting, abdominal pain, early satiety, and progressive weight loss [3,4]. Because these manifestations are nonspecific and often develop gradually, they may be attributed to more common gastrointestinal disorders, delaying the diagnosis. In the present case, persistent early satiety preceded the radiologic diagnosis by several years and resulted in marked weight loss despite preserved appetite.

The mechanism of obstruction differs from that of other causes of duodenal compression. In aortoduodenal syndrome, the aneurysm directly compresses the fixed third portion of the duodenum through mass effect [1,4]. In contrast, superior mesenteric artery syndrome results from compression of the third portion of the duodenum between the aorta and the superior mesenteric artery due to a reduced aortomesenteric angle and distance, typically in patients with significant weight loss [6]. Differentiating these entities is clinically important because they have distinct underlying mechanisms and therapeutic approaches. The key imaging and clinical differences are summarized in Table 1.

**Table 1:** Differential diagnosis of duodenal compression.

Feature	Aortoduodenal syndrome	Superior mesenteric artery syndrome	Neoplastic compression	Intrinsic duodenal disease
Etiology	Abdominal aortic aneurysm (AAA)	Loss of mesenteric fat → reduced aortomesenteric angle	Tumor (pancreatic, duodenal, and retroperitoneal)	Inflammatory, ulcerative, or fibrotic processes
Mechanism	Direct extrinsic compression (mass effect)	Vascular compression between aorta and SMA	Mass effect or infiltration	Luminal narrowing due to wall pathology
Typical age group	Older adults (≥60 y)	Young adults/cachectic patients	Variable (often older)	Variable
Clinical context	Known or incidental AAA	Significant weight loss, low BMI	Constitutional symptoms (weight loss and anemia)	Chronic GI symptoms
Aortomesenteric angle	Normal	Decreased (<25°)	Normal	Normal
Aortomesenteric distance	Normal	Decreased (<8-10 mm)	Normal	Normal
CT findings	AAA adjacent to D3 with extrinsic compression and proximal dilatation	Narrowed angle, reduced distance, compression of D3 without mass	Mass lesion causing displacement or infiltration	Wall thickening, mucosal irregularity
Key imaging feature	Presence of aneurysm causing compression	Reduced angle without mass	Solid or infiltrative lesion	Intrinsic duodenal abnormality
Response to treatment	Variable; may persist after EVAR	Often improves with nutritional therapy	Depends on tumor type	Depends on underlying disease

Contrast-enhanced CT is the imaging modality of choice because it simultaneously demonstrates the abdominal aortic aneurysm, the relationship between the aneurysm and the third portion of the duodenum, and the presence of upstream duodenal dilatation. Multiplanar reconstructions, particularly sagittal images, facilitate identification of the transition point and the mechanism of obstruction [1,2,4]. Although fluoroscopic upper gastrointestinal studies may demonstrate delayed transit and an abrupt cutoff at the third portion of the duodenum, contrast-enhanced CT remains the preferred imaging modality because it simultaneously depicts the vascular anatomy, the site of obstruction, and potential alternative diagnoses. Importantly, the diagnosis should not rely solely on the presence of an abdominal aortic aneurysm adjacent to the duodenum, as close anatomic proximity may also occur in the absence of clinically significant obstruction. Instead, proximal duodenal dilatation together with an abrupt transition point represents the most convincing imaging evidence of clinically significant obstruction.

One of the principal teaching points of this case is the temporal evolution of the imaging findings. Retrospective review of the earlier CT examinations demonstrated subtle anatomic proximity between the aneurysm and the third portion of the duodenum; however, convincing upstream duodenal dilatation was not present. These early imaging findings were insufficient to establish the diagnosis at the time but became clinically meaningful in retrospect after the development of overt proximal duodenal obstruction. Only the 2026 CT examination demonstrated the characteristic combination of proximal duodenal dilatation and an abrupt transition point, establishing the diagnosis of aortoduodenal syndrome. This observation emphasizes that isolated narrowing of the third portion of the duodenum may be nonspecific, whereas progressive upstream dilatation is the key imaging feature supporting clinically significant obstruction.

Management of aortoduodenal syndrome remains challenging. Open surgical repair has traditionally been considered the definitive treatment, whereas EVAR has emerged as a less invasive alternative in selected patients [4,7]. Nevertheless, symptom resolution after EVAR is not universal. In the present case, persistent symptoms after repair, together with the presence of an endoleak identified on postoperative imaging performed at the referring institution, raise the possibility that continued sac pressurization contributed to ongoing extrinsic compression despite aneurysm exclusion [5,8]. However, because detailed postoperative imaging and clinical follow-up from the referring institution were unavailable, this association should be regarded as hypothesis-generating rather than causal.

This case highlights the importance of integrating clinical evolution with serial imaging findings. In patients with abdominal aortic aneurysm and persistent upper gastrointestinal symptoms, careful evaluation of the relationship between the aneurysm and the third portion of the duodenum is warranted. Recognition of progressive proximal duodenal dilatation together with a transition point may facilitate earlier diagnosis and improve differentiation from other causes of proximal duodenal obstruction, particularly superior mesenteric artery syndrome.

## Conclusions

Aortoduodenal syndrome is an uncommon cause of proximal intestinal obstruction in patients with abdominal aortic aneurysm. Contrast-enhanced CT is essential for diagnosis by demonstrating the characteristic combination of proximal duodenal dilatation, an abrupt transition point, and extrinsic compression of the third portion of the duodenum. Recognition of these imaging findings in the appropriate clinical context may facilitate earlier diagnosis and improve patient management.

## Declaration of generative AI and AI-assisted technologies in the manuscript preparation process

During the preparation of this work, the author used OpenEvidence and OpenAI to assist with language refinement, structural organization of the manuscript, and editing for clarity. All scientific content, interpretation of imaging findings, literature review, and final manuscript decisions were performed and critically reviewed by the author, who takes full responsibility for the content of the published article.

## Patient consent

Written informed consent was obtained from the patient for publication of this case report and the accompanying images.
